# Predictive healthcare modeling for early pandemic assessment leveraging deep auto regressor neural prophet

**DOI:** 10.1038/s41598-024-55973-y

**Published:** 2024-03-04

**Authors:** Sujata Dash, Sourav Kumar Giri, Saurav Mallik, Subhendu Kumar Pani, Mohd Asif Shah, Hong Qin

**Affiliations:** 1 Nagaland University, Dimapur, 797112 Nagaland India; 2 Maharaja Srirama Chandra Bhanjadeo University, Baripada, 757003 Odisha India; 3 Department of Environmental Health, Harvard T H Chan School of Public Health, Boston, MA 02115 USA; 4 Krupajal Engineering College, Biju Patnaik University, Rourkela, India; 5 Kebri Dehar University, Kebri Dehar, Ethiopia; 6 Department of Computer Science and Engineering, University of Tennessee at Chattanooga, Chattanooga, USA

**Keywords:** Deep learning, Neural prophet, Auto-regressor network, Short-term forecasting, Lagged-regressor, Prophet, Neuroscience, Diseases, Neurology, Engineering

## Abstract

In this paper, NeuralProphet (NP), an explainable hybrid modular framework, enhances the forecasting performance of pandemics by adding two neural network modules; auto-regressor (AR) and lagged-regressor (LR). An advanced deep auto-regressor neural network (Deep-AR-Net) model is employed to implement these two modules. The enhanced NP is optimized via AdamW and Huber loss function to perform multivariate multi-step forecasting contrast to Prophet. The models are validated with COVID-19 time-series datasets. The NP’s efficiency is studied component-wise for a long-term forecast for India and an overall reduction of 60.36% and individually 34.7% by AR-module, 53.4% by LR-module in MASE compared to Prophet. The Deep-AR-Net model reduces the forecasting error of NP for all five countries, on average, by 49.21% and 46.07% for short-and-long-term, respectively. The visualizations confirm that forecasting curves are closer to the actual cases but significantly different from Prophet. Hence, it can develop a real-time decision-making system for highly infectious diseases.

## Introduction

The advancement in computing has facilitated the analysis of more complex and large datasets and triggered concern in data science and analytics. Computer science has paved the way with many sophisticated machine learning and deep learning methods that are receiving increased attention from decision-makers and forecasters^1^. In addition, statistical methods^2^, i.e., regression models, ARIMA, exponential smoothing, and Bayesian forecasting, have benefited from the advances in computing. Currently, the world is confronting several challenges and uncertainties, such as COVID-19, big storms, fires, international conflicts, social problems, etc., which warrant the requirement of effectual forecasting models to quantify these uncertainties. Similarly, many factors such as climate change, urbanization, and globalization cause the emergence of infectious diseases like COVID-19, which created havoc on a macro scale. Hence, regarded as a significant social problem^3^ that requires the identification of sensitive zones for the outbreak of the disease, and efficient forecasting tools are needed for the decision-making processes primarily for healthcare infrastructure planning, pharmaceutical supply chain management, and predicting the future incidence rate of epidemiology. Besides, COVID-19 does not seem to be a standalone crisis; there may be increasing chances^5^ of breaking pandemics soon that will undoubtedly force the healthcare system to work under a limited budget and frivolity, and the frequency of this situation will be more even in developed countries^4^.

There has been an enormous loss of human life, economic, and disorder of social life across the globe^4^ due to the uncontrolled spread of the pandemic. However, the rollout of the COVID-19 vaccine at the beginning of 2021 and the enforcement of the above restrictions have changed the course of the disease, death rate ebbed. The sudden spikes of cases during the fast-moving variant ‘Omicron’ were controlled by adding a booster to the vaccination, which helped in reducing the chance of hospitalization and death. There is a high likelihood that the new transmissible variants may renew the outbreak of the pandemic in the future^5^. Therefore, best-fitted mathematical models, such as ARIMA models^6^, have successfully forecasted the future daily cases for 90 days with 85% MAPE for the four worst-hit countries and four worst-hit states of India has helped for planning and management of healthcare systems and infrastructure. Researchers using SIR and SEIR mathematical models have predicted the reproduction parameter R0 for Indonesia^7^, which augmented the necessity of reliable forecasting models for predicting the early prevention of the pandemic. A deterministic model has been developed^8^ to study the interaction between HIV and tuberculosis to solve the nonlinear behavior of the parameters.

However, mathematical, and statistical forecasting models^9^ fail to capture the actual trend of the pandemic from the time-series analysis due to the limitations of being unable to handle large numbers of real-life parameters in a single model with the assumptions that they imposed on the model. The emergence of the deep learning model, RNN, efficiently handles problems involving time-series data. Long-short-term memory (LSTM), bi-directional LSTM (Bi-LSTM), and gated recurrent unit (GRU) are advanced RNN techniques used to overcome the vanishing gradient problem that is inherent to RNN. Another advantage of LSTM and Bi-LSTM is; that the former works in the future direction, and the latter works in both past and future directions. GRU has a simpler architecture without forget and update gates, unlike LSTM and Bi-LSTM. Three hybrid deep learning models combined with the Bayesian optimization method based on the multiple output forecasting strategies are proposed^10^ for both short-term and long-term forecasting. The Bayesian optimization method enhances the performance of the CNN, LSTM, and multi-head attention models, which is exhibited by evaluating the symmetric mean absolute percentage error (SMAPE) as 0.25 and 2.59 for short and long-term forecasting, respectively.

However, with massive time series data, neural network-based data-driven models have reclaimed their approval in forecasting. Nonetheless, the interpretability of the models remains an open research problem even after putting substantial efforts into pre-processing and hyperparameter tuning. However, the forecasting framework of Facebook Prophet^11^ has introduced explainability characteristics into the model. This is the first forecasting package that has popularized time-series forecasting and made it applicable to a wide demographic. Still, the limitations, i.e., lacking local context and extensibility, have restricted the adequacy of Prophet in healthcare and industrial applications. To overcome the above, an interpretable and user-friendly hybrid forecasting framework, NP^12^, combines the time-series components of Prophet with Neural Network (NN) modules such as auto-regression and covariates, which deal with non-linearity. Triebe et al.^12^ applied it to a set of synthetic datasets and observed that NP with auto-regression outperforms Prophet on all forecasting horizons. Most of the NP models reduced the forecast error by 50–90% for short to medium-range horizons and obtained improved accuracy for medium to large-range horizons. NP and LSTM-CNN^13^ enhance the seasonality analysis performance for a satellite and PV solar plant. A hybrid framework combining RNN and NP^14^ achieved better accuracy for channel predictors problem forecasting for a real-time dataset obtained from Nokia Bell-Labs. Default NP also has achieved the best forecasting performance for the COVID-19 problem^15^ compared to Random Forest and Poisson distribution models. Borges et al.^16^ applied the Prophet-LSTM hybrid model to forecast daily COVID-19 ICU entrances for a Brazilian city and found smaller values for MAEs compared to standalone models.

It is observed from the literature review that only a single paper^15^ has used the default NP model for forecasting the COVID-19 problem without considering the effect of individual components of the model. The model has overcome the limitations of the Prophet model by making it more approachable and effective for a wide demographic. The NP model combines the modules of Prophet that capture the linear relationship in the time series data with the Deep Neural Network (DNN) that captures the non-linear dynamics of the time series. This manuscript has proposed seven configurations of the hybrid NP model by using Deep-AR-Net model. Deep-AR-Net is designed to implement auto-regressor and lagged-regressor modules of NP. These models demonstrate the effect of components of the NP that address the limitations of the Prophet model. The experimental results quantify the performance of the individual interpretable forecast components. To optimize the forecasting performance, the hyper-parameters of the Prophet and neural network are tuned using the Grid search algorithm. Finally, the models’ performances are compared with Prophet and the default NP model to identify the best model for the problem. The proposed model is validated with COVID-19 time series datasets of five different countries that were affected badly by the recent third wave, namely, India, Germany, Iran, Mexico, and Spain, and lastly, identify the best enhanced NP model for each country for forecasting highly accurate confirmed cases for future reoccurrences. To the best of our knowledge, this is the first healthcare application where the interpretable, scalable and decomposition capabilities of the framework are demonstrated on COVID-19 time-series data. The objectives of the study are outlined below:

- Compared and studied the effect of individual components of Neural Prophet such as trend, seasonality, event, auto-regression, future regressor, and lagged regression with Prophet on the performances of forecasting of confirmed cases of India.
- Examined the effect of the performance of interpretable components, i.e., auto-regression and lagged regression of NP, on the combined forecast accuracy and prediction time by varying lags, horizons, hidden layers, and hidden neurons for short- and long-term forecasting models.
- Comparing forecasting performances of enhanced NP, and default NP models with the Prophet model to find the best model for COVID-19 time series data collected for five countries from 22nd January 2020 to 5th August 2022.

The remaining part of the paper is structured into three sections. “Proposed framework and methodology” illustrates the methodology implemented in the paper, and “Experimental setup, findings, and discussion” details the study's experiment findings and discussion. “Conclusion” concludes the study with future projections, followed by an exhaustive bibliography.

### Proposed framework and methodology

NeuralProphet, an extension of Facebook's Prophet, incorporates neural networks to enhance time series forecasting. While Prophet is effective in many forecasting scenarios, NeuralProphet has certain advantages that make it particularly suitable for epidemic forecasting such as:

(i) Complex Patterns Handling: Prophet relies on an additive model that includes components for trend, seasonality, and holidays. While effective, it may struggle with capturing highly complex patterns. On the other hand, the addition of neural networks allows for more flexibility in capturing intricate dependencies and non-linear patterns in epidemic data. (ii) Better Handling of Irregularities: Prophet is designed for datasets with regular patterns, Prophet might face challenges in handling irregularities and sudden changes in epidemic data whereas Neural networks excel in adapting to irregular patterns and abrupt changes, providing better adaptability to the dynamics of epidemic data. (iii) Feature engineering is crucial in Prophet to express patterns in terms of the provided components. However, Neural networks can automatically learn relevant features from the data, reducing the need for extensive manual feature engineering and making it more adaptable to diverse epidemic patterns. (iv) Non-linearity Handling: The additive model in Prophet may struggle with capturing complex non-linear relationships present in epidemic data, but Neural networks, by nature, are more adept at modeling non-linear relationships, enabling NP to better represent the intricacies of epidemic dynamics. (v) Customization with Neural Networks: Prophet is limited to its predefined components, which may not be sufficient for more complex epidemic scenarios while the incorporation of neural networks allows for customization and adaptability to a broader range of epidemic data characteristics.

In a nutshell, while Prophet is a powerful tool for various time series forecasting tasks, NP's integration of neural networks makes it particularly advantageous for capturing the nuanced and dynamic patterns often present in epidemic data. The ability to automatically learn complex features and adapt to irregularities positions NP as a valuable tool for epidemic forecasting.

**Figure 1:**
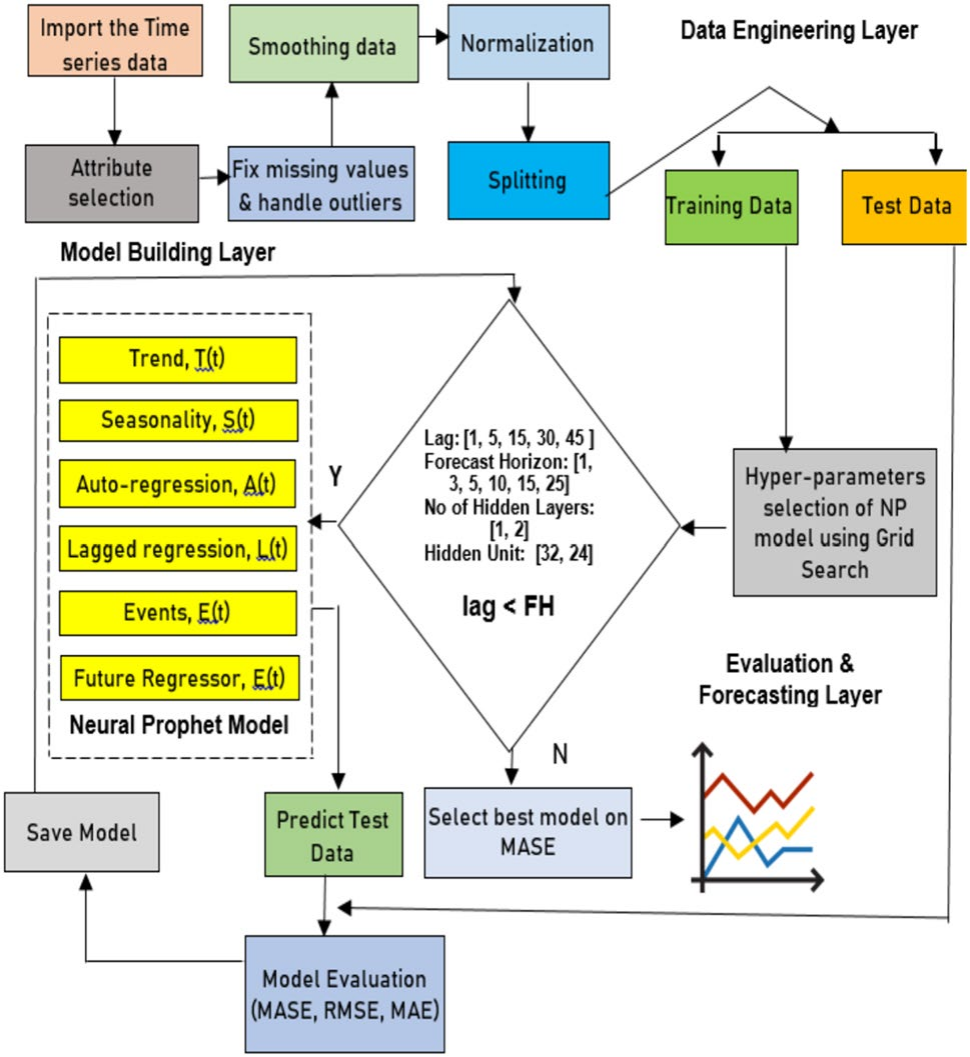
A framework of the proposed hybrid model.

This section describes the methodology used to achieve the objectives set by the proposed proposal. A framework is designed to show the procedure for accomplishing the targeted goals. Figure 1 depicts all framework components, i.e., the data engineering, model building, and evaluation and forecasting layers. The functioning of each layer is elaborated on below.

#### Data engineering layer

This layer explains data acquisition, selection, cleaning, smoothing, normalization, and splitting. COVID-19 time series data is collected for five countries from the Johns Hopkins University Coronavirus Data Stream^17^. CSV format was used to develop the dataset. The dataset description is illustrated in Table 1. Then a univariate time series is generated, selecting the confirmed case attribute of the pandemic.

**Table 1:** Time series dataset.

Dataset	Daily confirmed cases of COVID-19 pandemic for five countries (in .csv format)
Period	January 22, 2020, to August 5, 2022
Dimension	927 rows × 6 columns
Attributes	Date-reported, India, Germany, Iran, Mexico, Spain
Country with total confirmed cases	India: 4,41,26,994 Germany: 3,12,28,314 Iran: 74,31,485 Mexico: 68,21,746 Spain: 1,32,66,184

Then the dataset underwent cleaning by replacing the missing values with the mean of the seven days of consecutive data. Then, the data is smoothened by applying the 7-day moving average method. The time series sequences undergo a normalization process by applying the min–max given the range [0, 1]. Equation (1) shows the min–max expression.

1 $${{\text{x}}}_{{\text{scaled}}}=\frac{{\text{x}}-{{\text{x}}}_{{\text{min}}}}{{{\text{x}}}_{{\text{max}}}-{{\text{x}}}_{{\text{min}}}}$$

In this scenario, the test sequence used for predicting the confirmed cases gets de-normalized after the forecasting so that it will be like the actual time series test data. The time series contains 927 data points for each country, and out of this, 80% (742 days) of data is used for training keeping the rest 20% (185 days) for testing.

### Model building layer: proposed neural prophet model

Neural Prophet (NP)^12^ is a compartmentalized explainable model comprising six modules: trend (T), seasonality (S), event (E), future-regressor (FR), auto-regressor (AR), and lagged regressor (LR), each contributing an additive component to the forecast curve. All components can be configured independently and integrated to devise the model. Nevertheless, all six modules produce h outputs for h number of time steps to be predicted in the future and added up as $\widehat{{{\text{y}}}_{{\text{t}}}},\dots .,$ ${\widehat{{\text{y}}}}_{{\text{t}}+{\text{h}}-1}$ to the future values ${{\text{y}}}_{{\text{t}}}, \dots ,$ ${{\text{y}}}_{{\text{t}}+{\text{h}}-1}$. The full model is represented as represented in Eq. (2).

2 $$\widehat{{{\text{y}}}_{{\text{t}}}}=\mathrm{ T}\left({\text{t}}\right)+\mathrm{ S}\left({\text{t}}\right)+\mathrm{ E}\left({\text{t}}\right)+\mathrm{ FR}\left({\text{t}}\right)+\mathrm{ AR}\left({\text{t}}\right)+\mathrm{ LR}\left({\text{t}}\right)$$

The trend component is modelled as a continuous piece-wise linear series by combining an offset m with a growth rate k, allowing changes at various locations. The effect at time t1 is computed as shown in Eq. (3).

3 $${\text{T}}\left({\text{t}}\right)={\text{T}}\left({{\text{t}}}_{0}\right)+{\text{k}}\Delta {\text{t}}={\text{m}}+{\text{k}}\left({\text{t}}-{{\text{t}}}_{0}\right)$$

Thus, an interpretable nonlinear model is obtained for the trend module. The growth rate of the linear trend only varies for a finite number of changepoints n_C_, which is set to five in our model that corresponds to the five lockdowns imposed in India at different times. The set C can be defined as $\mathrm{C }= ({{\text{c}}}_{1},{\mathrm{ c}}_{2}, ..., {{\text{c}}}_{{\text{n}}})$. In between changepoints, the growth of the trend remained constant. The adjustment of the growth rate at each changepoint can be represented by a growth vector $\updelta \in {\mathbb{R}}^{{{\text{n}}}_{{\text{C}}}}$ and a corresponding offset vector $\uprho \in {\mathbb{R}}^{{{\text{n}}}_{{\text{C}}}}$. Another vector $\Gamma \left({\text{t}}\right) \in {\mathbb{R}}^{{{\text{n}}}_{{\text{C}}}}$ represents the status of time t concerning each changepoint. Hence, the trend T(t) at time t can be defined as represented in Eq. (4).

4 $$T\left({\text{t}}\right)=\left({\updelta }_{0}+\Gamma {({\text{t}})}^{{\text{T}}}\updelta \right).{\text{t}}+\left({\uprho }_{0}+\Gamma {({\text{t}})}^{{\text{T}}}\uprho \right)$$

where,

$$\updelta =\left({\updelta }_{1},{\updelta }_{2},{\updelta }_{3},\dots ,{\updelta }_{{{\text{n}}}_{{\text{C}}}}\right),\uprho =({\uprho }_{1},{\uprho }_{2},{\uprho }_{3},\dots ,{\uprho }_{{{\text{n}}}_{{\text{C}}}})$$

$$\Gamma \left({\text{t}}\right)=\left({\Gamma }_{1}\left({\text{t}}\right), {\Gamma }_{2}\left({\text{t}}\right), {\Gamma }_{3}\left({\text{t}}\right),\dots ,{\Gamma }_{{{\text{n}}}_{{\text{C}}}}\left({\text{t}}\right)\right),$$

$${\Gamma }_{{\text{j}}}\left({\text{t}}\right)=\left\{\begin{array}{c}1, \quad if\, t\ge {{\text{c}}}_{{\text{j}}}\\ 0, \quad otherwise\end{array}\right.$$

The Fourier series^18^ is used to model the periodic effect of yearly seasonal variations for this problem, represented in Eq. (5).

5 $${\text{s}}\left({\text{t}}\right)=\sum_{{\text{n}}=1}^{{\text{N}}}\left({{\text{a}}}_{{\text{n}}}{\text{cos}}\left(\frac{2\mathrm{\pi nt}}{{\text{P}}}\right)+{{\text{b}}}_{{\text{n}}}{\text{sin}}\left(\frac{2\mathrm{\pi nt}}{{\text{P}}}\right)\right)$$

where, the default value of P = 365.25 and N = 6.

The AR and LR modules are implemented by configuring an advanced AR-Net^19^ model named the Deep-AR-Net model. In contrast to classical AR, the Deep-AR-Net(p) can produce all h forecasts in a single model, which can be linear or non-linear depending on the complexity of the problem. It uses the last p observations of the target variables ${{\text{y}}}_{{\text{t}}-1},{{\text{y}}}_{{\text{t}}-2}, ... {{\text{y}}}_{{\text{t}}-{\text{p}}}$, as inputs and produces h-values corresponding to the AR-effect for each forecast step ${{\text{A}}}^{{\text{t}}} \left({\text{t}}\right), {{\text{A}}}^{{\text{t}}}\left({\text{t}}+1\right), \dots ..,{\mathrm{ A}}^{{\text{t}}}\left({\text{t}}+{\text{h}}-1\right)$. The module can be depicted as in Eq. (6).

6 $${{\text{A}}}^{{\text{t}}} \left({\text{t}}\right), {{\text{A}}}^{{\text{t}}}\left({\text{t}}+1\right), \dots ..,{\mathrm{ A}}^{{\text{t}}}\left({\text{t}}+{\text{h}}-1\right)={\text{Deep}}-{\text{AR}}-\mathrm{Net }\left({{\text{y}}}_{{\text{t}}-1}, {{\text{y}}}_{{\text{t}}-2}, \dots .,{\mathrm{ y}}_{{\text{t}}-{\text{p}}}\right)$$

We can have up to h different forecasts based on past predictions at any given moment. The AR module improves forecasting accuracy by using hidden layers in a NN, but interpretability may be compromised. To better understand our time series, we can use covariates or lagged regressors but can only rely on observed values up to t-1 during forecasting at time t. LR is represented in Eq. (7).

7 $${\text{L}}\left({\text{t}}\right)=\sum_{{\text{x}}\in {\text{X}}}{{\text{L}}}_{{\text{x}}}\left({{\text{x}}}_{{\text{t}}-1}, {{\text{x}}}_{{\text{t}}-2}, \dots , {{\text{x}}}_{{\text{t}}-{\text{p}}}\right)$$

To analyze the impact of each covariate in a set ${\text{X}}\in {\mathbb{R}}^{{\text{T}}\times {{\text{n}}}_{{\text{l}}}}$, we create a separate LR module for each m covariate x of length T. This allows us to determine the effect of each covariate on predictions individually. The module uses the last p observations of the covariate X as inputs and produces h additive components ${{\text{L}}}_{{\text{x}}}^{{\text{t}}}\left({\text{t}}\right), {{\text{L}}}_{{\text{x}}}^{{\text{t}}}\left({\text{t}}+1\right), \dots {{\text{L}}}_{{\text{x}}}^{{\text{t}}}\left({\text{t}}+{\text{h}}-1\right)$ for overall forecasts ${\widehat{{\text{y}}}}_{{\text{t}}}, {\widehat{{\text{y}}}}_{{\text{t}}+1}, \dots ,{\widehat{{\text{y}}}}_{{\text{t}}+{\text{h}}-1}$ as shown in Eq. (8).

8 $${{\text{L}}}_{{\text{x}}}^{{\text{t}}}\left({\text{t}}\right), {{\text{L}}}_{{\text{x}}}^{{\text{t}}}\left({\text{t}}+1\right), \dots {{\text{L}}}_{{\text{x}}}^{{\text{t}}}\left({\text{t}}+{\text{h}}-1\right)={\widehat{{\text{y}}}}_{{\text{t}}}, {\widehat{{\text{y}}}}_{{\text{t}}+1}, \dots ,{\widehat{{\text{y}}}}_{{\text{t}}+{\text{h}}-1}$$

The lag value is chosen as twice the value of horizon. Seven different configurations are used here for the Deep-AR-Net model.

1. The first model is devised by considering the default Deep-AR-Net configuration, a single-layer neural network with p and h I/O without bias and activation function. Hence, the model can be defined as a multiplication of a vector–matrix ***y* = *Wx*** for producing the predicted AR results ***y Є R***^***h***^ for the lagged information as input ***x Є R***^***p***^***.***
2. Next, five interpretable Deep-AR-Net models are configured for five sets of lagged observations for p = 1, 5, 15, 30, 45, and each model predicts the single and multi-step forecast horizon with h = 1, 3, 5, 10, 15, 25. No significant improvement in the accuracy of the metrics are observed when p > 45 and h > 25.
3. Lastly, Deep-AR-Net is used to devise non-linear models by adding hidden layers. The two best models with 32 and 24 hidden neurons, i.e., (1 × 32 NN) and (2 × 24 NN), are chosen from the simulation. Each hidden layer has used a rectified linear unit (ReLU) as an activation function. The output layer produces h outputs without using activation function and bias. Therefore, the model with *ℓ* hidden layers and *d* dimension is represented in Eqs. (9)–(11).
  9 $${{\text{a}}}_{1}={{\text{f}}}_{{\text{a}}}\left({{\text{W}}}_{1}{\text{x}}+{{\text{b}}}_{1}\right)$$
  10 $${{\text{a}}}_{{\text{i}}}={{\text{f}}}_{{\text{a}}}\left({{\text{W}}}_{{\text{i}}}{{\text{a}}}_{{\text{i}}-1}+{{\text{b}}}_{{\text{i}}}\right)\mathrm{ \,for\, i }\in \left[2, \dots ,{\text{l}}\right]$$
  11 $${\text{y}} = {\text{W}}_{\ell + 1} {\text{a}}_{\ell }$$

where

$${{\text{f}}}_{{\text{a}}}\left({\text{x}}\right)={\text{RELU}}\left({\text{x}}\right)=\left\{\begin{array}{c}x, x\ge 0\\ 0, x<0\end{array}\right.$$

The event module is computed considering the five lockdown periods imposed by the government of India during the pandemic. The default value is used for other countries to compute the event module. The models are trained with 80% of the time series data and predicted with the remaining 20% of the data. However, the hyperparameters have a significant influence on the model. The grid search algorithm is employed here for optimizing the NP and Prophet parameters.

The models were optimized using training data from Jan 2020 to Feb 2022 (742 days) and tested on data from Feb to Aug 2022 (185 days). AdamW^12^ was used for optimization and Huber loss^12^ for default loss function. Training was fine-tuned with momentum β at 0.9, weight decay at 1e-04, learning rate at 0.001, and 200 epochs.

#### Evaluation and forecasting layer

The Prophet^11^ model is used here as a benchmark to be compared with Deep-AR-Net enhanced Neural Prophet models. The hyperparameters, namely, number of lags p, forecasting horizons h, number of hidden layers and hidden neurons, learning rate, and configurations of Deep-NN, are experimentally set to quantify the susceptibility of NP while choosing the parameters depicted in Table 2. For the empirical evaluation of the COVID -19 confirmed cases of five countries, the horizon is set to [∞, 1, 3, 5, 10, 15, 25], where ∞ is used to forecast the test time series of Prophet and default NP set as a whole.

**Table 2:** Hyper-parameters of Prophet model.

Hyper-parameter	Value	Method of selection
n_forecasts	[∞, 1, 3, 5, 10, 15, 25]	Iteratively
n_lags	[1, 5, 15, 30, 45]	Iteratively
Num_hidden_layers	[1, 2]	Iteratively
Learning_rate	0.001	Grid search optimization
Momentum	0.9	Grid search optimization
Epochs	200	Grid search optimization
Loss_func	Huber	Triebe et al.^12^
Optimizer	AdamW	Triebe et al.^12^
n_changepoints	5	Grid search optimization
Changepoint_range	[0.8–0.9]	Grid search optimization
Changepoint_prior_scale	[0.01–0.05]	Grid search optimization
Seasonality_prior_scale	[0.01–0.03]	Grid search optimization
Weekly seasonality	False	Dash et al.^11^
Monthly seasonality	False	Taylor et al.^18^
Quarterly seasonality	False	
Yearly seasonality	True	
Holidays	None	

For measuring the effectiveness of the proposed models, three statistical metrics, Mean Absolute Error (MAE), Mean Absolute Scaled Error (MASE), and Root Mean Square Error (RMSE), are employed. MASE, Eq. (12) evaluates the model’s performance compared with Naïve forecasting, and a smaller value implies an enhancement over the Naïve. It is also independent of the scale of the forecast. The lower the value of MAE in Eq. (13), MASE, and RMSE in Eq. (14), the higher the forecasting performance. The mathematical expressions of the metrics are as follows:

12 $${\text{MAE}}=\frac{\sum_{{\text{i}}=1}^{{\text{J}}}\left|{{\text{y}}}_{{\text{i}}}-{\widehat{{\text{y}}}}_{{\text{i}}}\right|}{{\text{J}}}$$

13 $${\text{MASE}}=\frac{\frac{1}{{\text{J}}}\sum_{{\text{j}}={\text{T}}+1}^{{\text{T}}+{\text{J}}}\left|{{\text{y}}}_{{\text{i}}}-{\widehat{{\text{y}}}}_{{\text{i}}}\right|}{\frac{1}{{\text{T}}-1}\sum_{{\text{i}}=2}^{{\text{T}}}\left|{{\text{y}}}_{{\text{i}}}-{{\text{y}}}_{{\text{i}}-1}\right|}$$

14 $${\text{RMSE}}=\sqrt{\frac{\sum_{{\text{i}}=1}^{{\text{J}}}{\left({{\text{y}}}_{{\text{i}}}-{\widehat{{\text{y}}}}_{{\text{i}}}\right)}^{2}}{{\text{J}}} }$$

where T is the size of train data, J, size of test data, ${{\text{y}}}_{{\text{i}}}$ is the actual or observed value, and ${\widehat{{\text{y}}}}_{{\text{i}}}$ is the predicted value.

## Experimental setup, findings, and discussion

The proposed framework is implemented using Python 3.8 version in Jupyter Notebook and executed in Windows 11, Intel(R), core-i7-7500U CPU @2.80 GHz and 16.0 GB RAM. The packages used for prediction and graphical presentation of the findings are as follows: NumPy, Pandas, FBprophet, NeuralProphet, Sklearn, seaborn, and matplotlib. The following experiments are carried out for all four countries to predict the incidence rate, but due to space limitation, all results are provided for India, and only the last two results are furnished for the remaining four countries. The conducted experiments are the following:

1. Illustration of the accuracy of explainable modular components- with and without lag for India.
2. Comparison of training and prediction computing time for all the countries.
3. Benchmark results for India for all three metrics using Eqs. (12), (13) and (14) and the results based on MASE errors are furnished across remaining four countries. Further results are furnished by introducing NN components.
4. Comparison of forecasting curves of NP model with Prophet model.

### Illustration of results of explainable decomposed components

**Figure 2:**
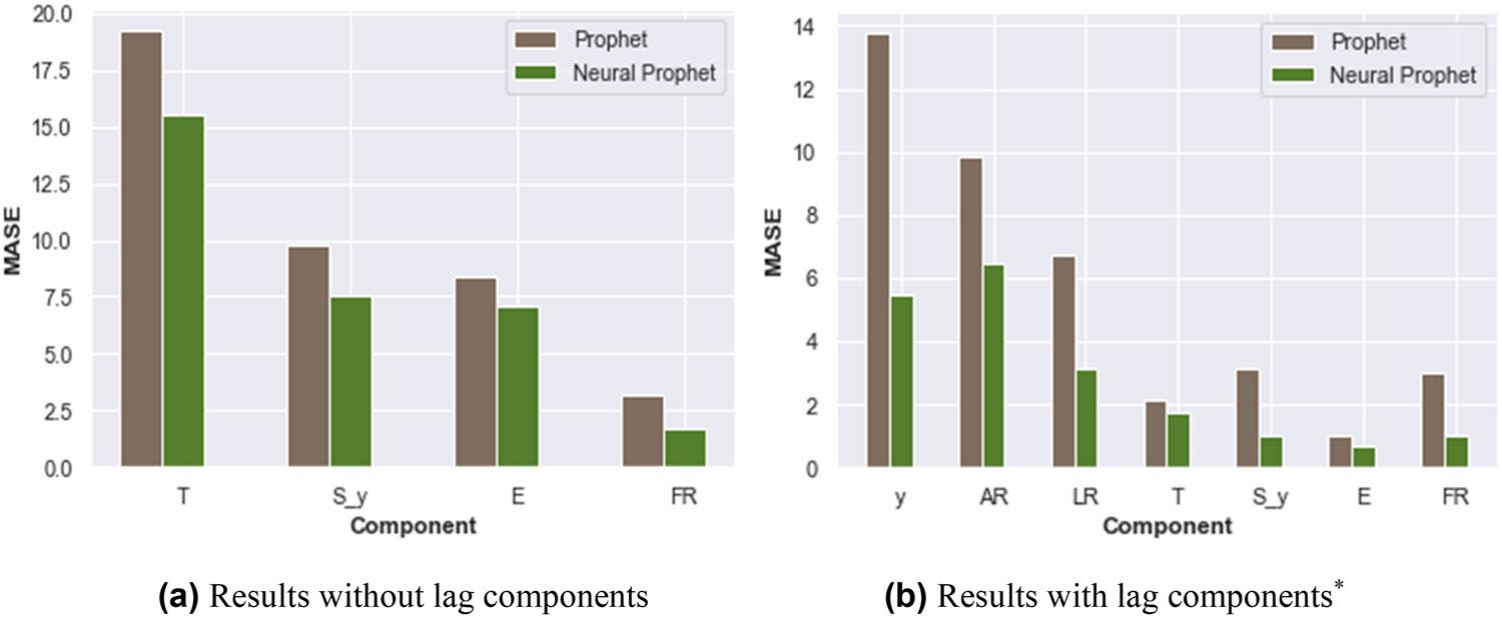
Comparing component-wise MASE of NP with the actual component value prophet on confirmed time series data of India. (**a**) Results without lag components. (**b**) Results with lag components*. *T* trend, *S_y* yearly seasonal effect, *E* event, *FR* future regressor, *y* sum of the components, *AR* auto-regression, *LR* lagged regressor. *Default value of prophet considered for AR and LR as both do not belong to prophet.

The experimental results of all the components of Prophet and NP are displayed separately for without and with lag features in Fig. 2a,b, respectively. Figure 2a, NP without lag components shows a significant performance of MASE by reducing all four-component errors by 19.2%, 23.5%, 15.5%, and 46.9% compared to Prophet. The most significant difference observed in FR component of NP is 46.9%.

NP models with lag components exhibit a significant enhancement while decomposing lag regressors. The AR and LR components show significantly better accuracy compared to Prophet’s zero prediction. Figure 2b with lag components demonstrates an outstanding performance of MASE for all the components of NP, reducing the error by 60.4% for y-component, 34.7% for AR, 53.4% for LR, 34.7% for T, 67.3% for S_y, 32.7% for E and 65.1% for FR. This analysis shows accuracy of the decomposed components is an appropriate measure of the performance of the forecasting model which generalizes effectively by capturing the underlying changes^19^.

#### Training and prediction time

Table 3 shows NP takes almost 4.0 times more training than Prophet. Both show a vast difference in computing time for training while the prediction time shows the reverse relationship. The prediction time of NP is significantly faster by 1.9 times in average compared to Prophet. This finding justifies NP requires some additional resources to fit in the model while needs significantly fewer resources for prediction. However, the faster prediction time of NP indicates that it can be deployed for any time-sensitive most likely healthcare application. It typically requires a dependable computation of the following prediction in a fraction of a second.

**Table 3:** Computing time in seconds for training and prediction with deep-AR-net enhanced NP components (NP*).

Model/country	Training/prediction time				
	India	Germany	Iran	Mexico	Spain
Prophet	2.07/1.05	3.04/1.54	2.01/0.99	2.88/1.23	2.06/1.07
NP*	7.78/0.86	7.87/0.89	6.87/0.46	5.89/0.57	4.21/0.43

#### Benchmark results based on MASE using the deep-AR-net

The results of the forecast accuracy in MASE, MAE and RMSE are exhibited in Table 4 for India. The analysis of the results highlights the following observations. First, both frameworks in their default mode performs nearly similar and significantly inferior compared to Naïve one-step forecast horizon. Second, for a higher number of lags, a substantial improvement in the forecast of NP is observed and highlighted in bold. Also, they perform consistently better than the Naïve one-step horizon. Hence, a greater number of lags pave the way to better performance. Third, for multi-step forecast horizons, a consistent increase of performance is observed for NP across all metrics. Therefore, NP with any number of lags accomplishes better than Prophet and default NP. Four, the analysis of the impact of different NN configurations on forecasting performs exceptionally better than the linear NP model and Prophet model.

**Table 4:** Performance of enhanced NP model based on MAE Value (in K) for different forecast horizons for India.

Models	Forecast horizons					
	1	3	5	10	15	25
Prophet	43.7					
Default NP	39.9					
NP* 1 lag	4.9	NA	NA	NA	NA	NA
NP* 5 lags	4.2	6.1	NA	NA	NA	NA
NP* 15 lags	3.0	3.4	6.0	10.1	NA	NA
NP* 30 lags	2.7	3.7	5.2	11.3	15.2	NA
NP* 45 lags	1.5	3.3	4.9	12.2	19.1	28.3
NP* 45 lags, 1 × 32	1.9	3.9	3.6	9.8	17.9	22.3
NP* 45 lags, 2 × 24	**1.7**	**3.4**	**3.9**	**8.9**	**16.9**	**21.5**

Nevertheless, the MASE error is considered a dependable metric as it normalizes the error into a standardized space for comparing the performance of other models configured for India. Therefore, MASE error is used to compare the forecast performances of different models for best lag with different horizons across different countries displayed in Table 7, highlighting best performances in bold. From Tables 4, 5, 6, and 7, it is evident that 45 lags for India, 30 lags for Germany, Mexico, and Spain, and 15 lags for Iran for 1-step reduces the MASE error by 54.7% for short term forecasting and for long term 45lags with 2 × 24 NN components for 25 steps reduces the error by 22% for all five countries. Hence, 45lags with 2 × 24 NN components for 25 steps is considered the best combined model for forecasting the confirmed cases for all the countries. This analysis is crucial because the number of lags determines how historical data is considered, which affects the accuracy of the forecasting. On the other hand, the neural network configuration impacts the model's complexity and learning ability, which, in turn, affects its capability to recognize patterns.

**Table 5:** RMSE value (in K) for different forecast horizons for India.

Models	Forecast horizons					
	1	3	5	10	15	25
Prophet	43.7					
Default NP	39.9					
NP* 1 lag	5.9	NA	NA	NA	NA	NA
NP* 5 lags	4.5	7.4	NA	NA	NA	NA
NP* 15 lags	3.5	5.9	6.9	12.2	NA	NA
NP* 30 lags	4.4	4.4	6.4	14.4	15.2	NA
NP* 45 lags	3.1	4.2	5.9	14.6	22.6	34
NP* 45 lags, 1 × 32	3.7	5.3	4.8	13.8	19.8	29.9
NP* 45 lags, 2 × 24	**3.7**	**4.9**	**4.9**	**13.1**	**18.9**	**26.9**

**Table 6:** MASE Value for different forecast horizons for India.

Models	Forecast horizons					
	1	3	5	10	15	25
Prophet	18.43					
Default NP	15.77					
NP* 1 lag	2.56	NA	NA	NA	NA	NA
NP* 5 lags	2.15	3.15	NA	NA	NA	NA
NP* 15 lags	1.52	1.77	3.09	5.35	NA	NA
NP* 30 lags	1.33	1.85	2.59	5.35	8.07	NA
NP* 45 lags	1.14	1.16	2.38	6.08	9.82	9.14
NP* 45 lags, 1 × 32	1.17	1.34	1.97	3.76	6.87	7.21
NP* 45 lags, 2 × 24	**1.16**	**1.29**	**1.98**	**3.18**	**6.83**	**7.13**

**Table 7:** MASE Value for different forecast horizons for India, Germany, Iran, Mexico and Spain.

Model specification	India	Germany	Iran	Mexico	Spain
Any step ∞					
Prophet	18.43	17.98	11.65	15.43	16.87
Default NP	15.77	13.76	10.97	11.23	10.98
NP*1 step					
NP* (1lag)	2.56	6.76	1.98	2.54	4.97
NP* (5 lags)	2.15	4.43	1.87	2.04	3.99
NP* (15 lags)	1.52	3.54	**1.43**	1.97	2.76
NP* (30 lags)	1.33	**2.98**	1.65	**1.43**	**1.07**
NP* (45 lags)	1.14	3.65	1.69	1.87	1.98
NP* (45lags, 1 × 32NN)	1.17	3.27	1.53	1.65	1.71
NP* (45 lags, 2 × 24 NN)	1.16	3.16	1.52	1.56	1.43
3 steps					
NP* (5 lags)	3.15	6.98	2.83	3.91	6.98
NP* (15 lags)	1.77	4.34	1.75	1.87	4.09
NP* (30 lags)	1.85	**3.17**	2.09	1.65	3.11
NP* (45 lags)	1.16	3.88	2.34	1.97	3.98
NP* (45 lags, 1 × 32 NN)	1.34	3.27	1.98	1.88	4.12
NP* (45 lags, 2 × 24 NN)	1.29	3.21	1.83	1.81	4.99
5 steps					
NP* (15 lags)	3.09	7.54	3.82	4.34	7.87
NP* (30 lags)	2.56	5.34	2.45	3.08	5.98
NP* (45 lags)	2.38	4.56	2.02	2.97	4.65
NP* (45 lags, 1 × 32 NN)	1.97	3.99	1.34	2.99	3.09
NP* (45 lags, 2 × 24 NN)	1.98	4.02	1.36	2.98	3.98
10 steps					
NP* (15 lags)	5.35	8.76	6.78	8.76	9.73
NP* (30 lags)	5.35	7.86	5.87	7.65	7.65
NP* (45 lags)	6.08	5.45	4.34	5.45	6.98
NP* (45 lags, 1 × 32 NN)	3.76	4.64	3.90	4.34	4.03
NP* (45 lags, 2 × 24 NN)	3.18	3.68	1.67	2.14	3.01
15 steps					
NP* (30 lags)	8.07	11.34	9.81	11.76	12.09
NP* (45 lags)	9.82	10.32	9.09	9.91	10.14
NP* (45 lags, 1 × 32 NN)	6.87	8.97	6.73	7.06	9.07
NP* (45 lags, 2 × 24 NN)	6.83	7.98	6.01	5.96	6.34
25 steps					
NP* (45 lags)	9.14	12.45	11.84	13.43	16.76
NP* (45 lags, 1 × 32 NN)	7.21	10.98	8.08	11.09	11.27
NP* (45 lags, 2 × 24 NN)	7.13	9.87	5.67	7.61	8.43

In the real world, the sensitivity of a model can affect its ability to adapt to changing pandemic dynamics. If a model has too few lags, it may underfit and miss important trends. On the other hand, if it has too many lags, it may introduce noise. An improper neural network configuration can result in overfitting or underfitting, which may hinder generalization.

To reduce the impact of these effects, it is important to adopt a flexible approach to selecting the time lag and to fine-tune the parameters of the neural network based on ongoing data analysis. To keep the model up to date in a dynamic healthcare environment, it is crucial to continuously monitor and adjust it.

#### Comparison of forecasting curves

**Figure 3:**
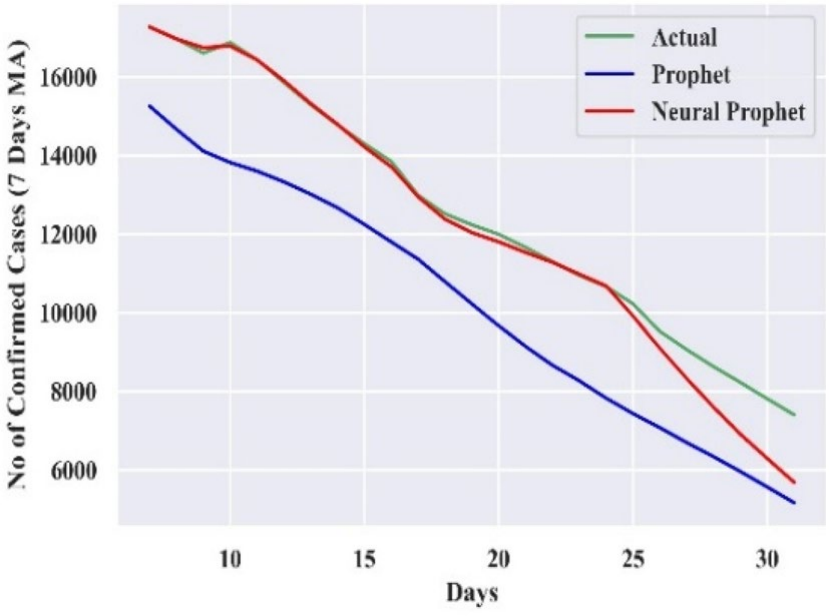
Forecasting curve for India.

**Figure 4:**
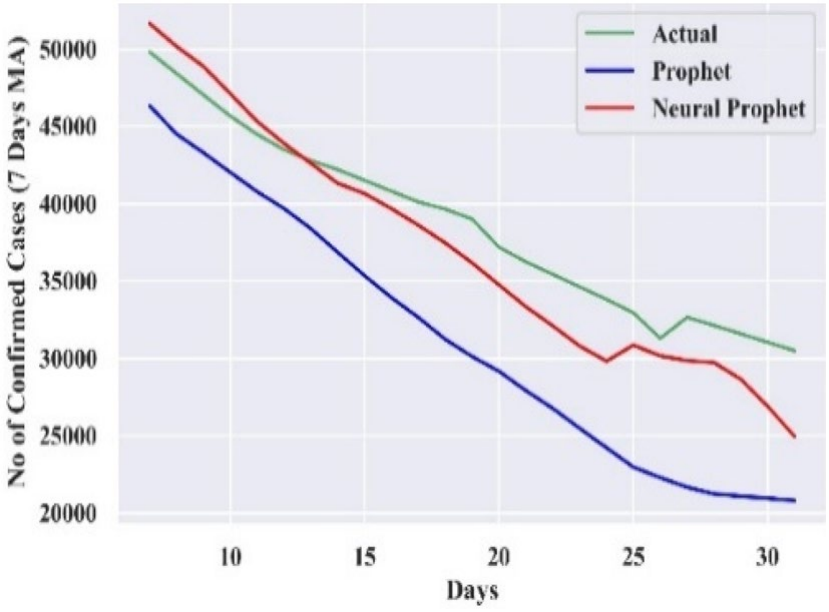
Forecasting curve for Germany.

**Figure 5:**
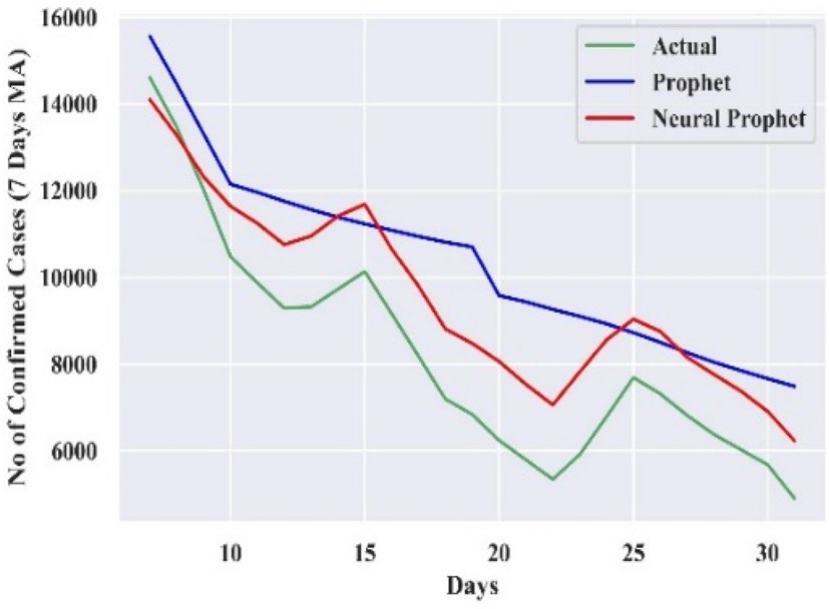
Forecasting curve for Iran.

**Figure 6:**
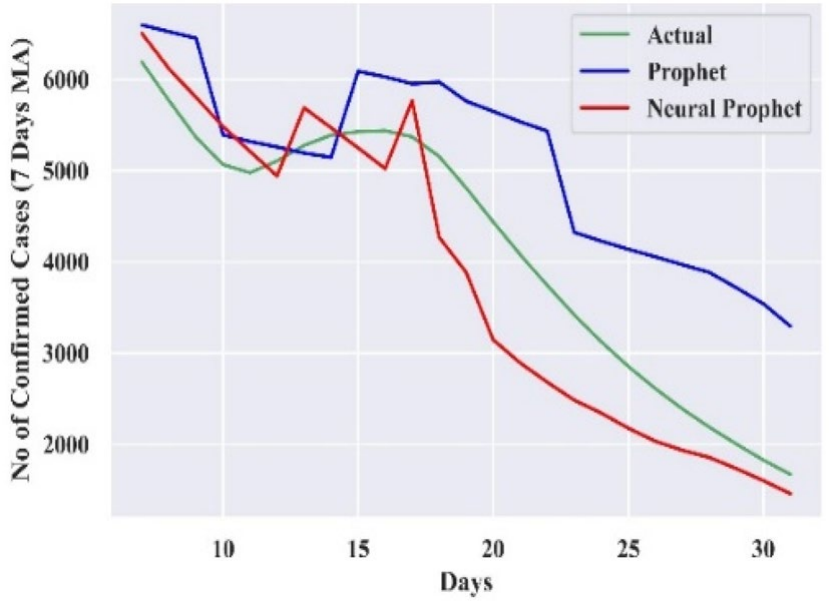
Forecasting curve for Mexico.

**Figure 7:**
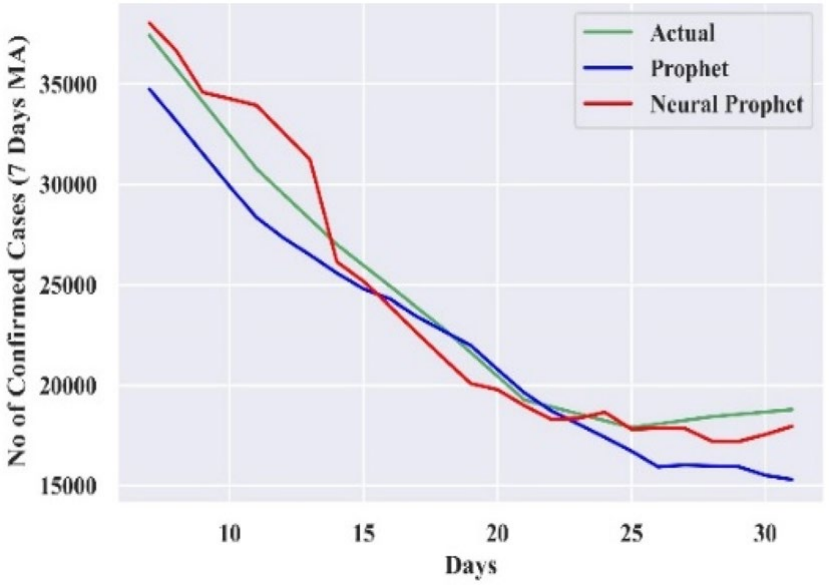
Forecasting curve for Spain.

The performance of the best-combined forecast model for all the countries is compared with actual cases and Prophet, which is displayed in Figs. 3, 4, 5, 6, and 7. The curves support the experimental analysis. The Deep-AR-Net models enhance the efficiency of the combined forecast for the short to medium-range forecast horizons evidenced by Tables 4, 5, 6, and 7. The MASE forecast error is reduced on average by 49.21% for all the countries for the short-to-medium range. Hence, the models help NP overcome the “local context” limitation of the Prophet. Similarly, the Deep-AR-Net model enhances NP’s forecast accuracy by reducing the average MASE for the medium-to-long range by 46.07%. The accuracy would be higher if data were available for four to five years. The critical observations of this experiment are that the number of lags and Deep-NN parameters significantly impact forecast accuracy. Models with a higher number of lags perform better. So, the models are sensitive to the optimal choice of parameters.

The forecasting curves for pandemic forecasting significantly differ between the NP and Prophet models. These differences are crucial for decision-making in public health, resource allocation, and policy planning. The accuracy variations determine the preference for a more reliable forecasting model. Understanding these disparities provides valuable insights into the strengths and weaknesses of each model, thereby helping researchers, policymakers, and healthcare professionals choose the model that best aligns with data characteristics and specific contexts. The practical significance of these differences extends to resource optimization, which favours models with lower computational requirements or quicker predictions when computational efficiency is crucial. In summary, these differences impact decision-making, model selection, and resource allocation in managing and mitigating the impact of a pandemic.

## Conclusion

This paper presents a forecasting framework called NP, based on a hybrid modular approach developed by Facebook Prophet. The framework has been customized by incorporating domain knowledge of the disease to forecast confirmed cases of COVID-19 across five different countries accurately. The hyperparameters of the six modular components of NP are optimized using the Grid Search algorithm. The Deep-AR-Net model utilizes both AR and LR components of NP, resulting in improved interpretability and enabling multivariate and multi-step forecasting. By enhancing the combined effects of the remaining components, this model improves the forecasting of confirmed cases. Empirical results demonstrate the success in achieving the objectives of the problem. Our analysis found that the Deep-AR-Net enabled NP and default NP models perform better than Prophet across all forecast horizons. Additionally, this component has increased the model's interpretability and enabled it to predict short-term future cases and medium to long-range horizons, as evidenced by the MASE values. The Deep-AR-Net-based NP models have reduced forecast error by 45% to 71% on short to medium-range horizons. We have also observed that the forecasting curves are closer to the actual cases but differ significantly from the Prophet curve for all five countries. It's worth noting that the performance of NP models depends on the number of lags and Deep-AR configuration. However, the advanced features of NP empower forecasting researchers with an explainable and scalable framework that can handle all types of healthcare applications. This model can aid in early detection and diagnosis, but there are limitations due to data size and training time. Comparative studies with the same datasets are important to evaluate the efficiency of NeuralProphet in comparison to other models. They provide valuable insights into its computational complexity and effectiveness. Future work can use efficient optimization algorithms to improve training time and create hybridized models combining RNN and Prophet.

## Data Availability

The datasets used and/or analysed during the current study available from the corresponding author on reasonable request.
